# Nerve Response to Superelastic Shape Memory Polyurethane Aerogels

**DOI:** 10.3390/polym12122995

**Published:** 2020-12-15

**Authors:** Martina Rodriguez Sala, Omar Skalli, Nicholas Leventis, Firouzeh Sabri

**Affiliations:** 1 Department of Physics and Materials Science, University of Memphis, Memphis, TN 38152, USA; 2 Department of Biological Science, University of Memphis, Memphis, TN 38152, USA; oskalli@memphis.edu; 3 Department of Chemistry, Missouri University of Science and Technology, Rolla, MO 65409, USA; nleventis@aerogel.com

**Keywords:** aerogel, scaffold, PC12 neuronal cells, superelastic shape memory polyutherane aerogel, topography, stiffness

## Abstract

We have previously shown the suitability of aerogels as scaffolds for neuronal cells. Here, we report on the use of superelastic shape memory polyurethane aerogels (SSMPA). SSMPA have a distinctly different stiffness than previously reported aerogels. The soft and deformable nature of SSMPA allowed for radial compression of the aerogel induced by a custom designed apparatus. This radial compression changed the pore diameter and surface roughness (Sa) of SSMPA, while maintaining similar stiffness. Two varieties of SSMPA were used, Mix-14 and Mix-18, with distinctly different pore diameters and Sa. Radial compression led to a decreased pore diameter, which, in turn, decreased the Sa. The use of custom designed apparatus and two types of SSMPA allowed us to examine the influence of stiffness, pore size, and Sa on the extension of processes (neurites) by PC12 neuronal cells. PC12 cells plated on SSMPA with a higher degree of radial compression extended fewer neurites per cell when compared to other groups. However, the average length of the neurites was significantly longer when compared to the unrestricted group and to those extended by cells plated on SSMPA with less radial compression. These results demonstrate that SSMPA with 1.9 µm pore diameter, 1.17 µm Sa, and 203 kPa stiffness provides the optimum combination of physical parameters for nerve regeneration.

## 1. Introduction

Cells are surrounded by an intricate and ever-changing microenvironment that includes the extracellular matrix (a complex heterogeneous 3-D network of extracellular proteins and glycoproteins), neighboring cells and signaling molecules. Within this environment, cells are subjected to mechanical forces that result from the stiffness and porosity of the extracellular matrix, body motions and interactions with the surrounding environment [1]. The different components of the cell surroundings affect cell behavior, such as adhesion to the substrate, cell morphology, differentiation, collective organization with tissues, etc. [2]. Recent efforts have focused on devising materials suited to guide cellular behavior to fit specific biomedical applications. The physical properties and microstructure of biomaterials, such as stiffness, topography, porosity and size scale, are key to guide cell behavior in a controlled manner [1]. Both the topography and stiffness of biomaterials have been shown to greatly impact cell behavior by influencing a variety of cell functions, such as stem cell differentiation [3,4], cell migration, spreading and shape [5], stimulation of osteoblasts promoting bone regeneration [6], etc. Biomaterials have been designed with specific physical properties and microstructures to understand how these parameters influence cell behavior. The surface topography of biomaterials can include grooves, grids and pillars [7,8,9]. However, these topographies differ from those found in tissues, and, therefore, it is unclear if they are relevant to clinical applications.

Aerogels have been proven to be biocompatible materials that can be used for medical applications, such as cardiovascular implantable devices [10] and implants for nerve repair [11,12,13,14]. In vitro experiments with cultured cells have also shown that aerogels can influence cell properties, such as the extension of neurites by neuronal cells [15,16,17]. The potential of aerogels as scaffolds for tissue engineering is due to their high porosity, good mechanical strength and biocompatibility [18,19,20]. Moreover, aerogel properties can be modified and tuned for specific biomedical applications [21,22]. The ability to tune aerogels’ properties enables a wide range of aerogel types, differing in their surface topography and stiffness [13,23,24], to be synthesized. The outcome of peripheral nerve repair is frequently poor, with a low percentage of patients recovering sensibility and motor function. Ongoing efforts aim to improve current approaches to nerve repair to result in better sensibility and motor recovery [25]. We have previously reported that aerogels promote the extension of long neurites by neuronal cells and that this effect is increased by subjecting cells grown on aerogels to an electric field [12,13,23,24].

In this study, we used superelastic shape memory polyurethane aerogels (SSMPA), which comprise a recently developed soft and deformable type of polyurethane aerogel [26,27,28,29] with distinctly different stiffness characteristics than previously studied polyurethane aerogels [30,31] or aerogels used as scaffolds for neuronal cells [12,13]. All SSMPAs are linearly elastic materials, allowing for radial compression while the stiffness remains practically constant. However, two specific types of SSMP aerogels were chosen for this study, and, for cross-reference purposes with previous work [27], they are referred to as Mix-14 and Mix-18; that choice was based on the similar densities and porosities of the two types of aerogels (Mix-14: bulk density of 0.529 ± 0.009 g cm^−3^, porosity of 56.1 ± 0.8 *v*/*v*; Mix-18: bulk density of 0.506 ± 0.006 g cm^−3^ and porosity of 57.7 ± 0.5 *v*/*v*) but primarily on their identical bicontinuous morphologies, despite their different chemical compositions [27]. Radial compression was carried out using a custom designed restrictor apparatus, inducing changes in the pore diameter and the Sa of the materials. At that point, the interaction of PC12 neuronal cells with the surface of the SSMPA at different stages of radial compression was investigated. The cellular response to the two types of SSMPA chosen for this study, Mix-14 and Mix-18, at different radial compression states was quantified as a function of pore size and surface topography to further understand the role of surface topography in guiding the extension of neurites.

## 2. Materials and Methods

### 2.1. Superelastic Shape Memory Polyurethane Aerogels (SSMPA)

Superelastic shape memory polyurethane aerogels (SSMPA) designated as Mix-14 and Mix-18 were prepared before as part of a broader Design-of-Experiments (DoE) statistical study, the objective of which was to probe the shape memory effect in poly(isocyanurate-polyurethane) aerogels (PIR-PUR aerogels) as a function of the chemical composition [27]. The prefix “Mix” refers to the fact that the DoE study utilized PIR-PUR formulations, where the polyurethane (PUR) part consisted, for the most part, of mixed alcohols, as opposed to exclusively single-alcohol formulations that had been described before [28,29]. Thus, Mix-14 was based on a high-concentration sol (25% *w*/*w* of monomers) that included only triethylene glycol (TEG) as a diol for PUR, while Mix-18 was based on a mixture of three diols (diethyene glycol, trietheylene glycol and tetraethylene glycol). In both Mix-14 and Mix-18, the isocyanurate (PIR) part of the PIR-PUR was introduced as part of an aliphatic triisocyanate (Desmodur N3300A [31]). Wet gels of both Mix-14 and Mix-18 were prepared in CH_3_CN/acetone mixtures (7:1 *v*/*v*) at room temperature (RT) using dibutyltin dilaurate as the catalyst. Further synthesis details are given in Reference [27].

### 2.2. Compression of SSMPA

**Figure 1:**
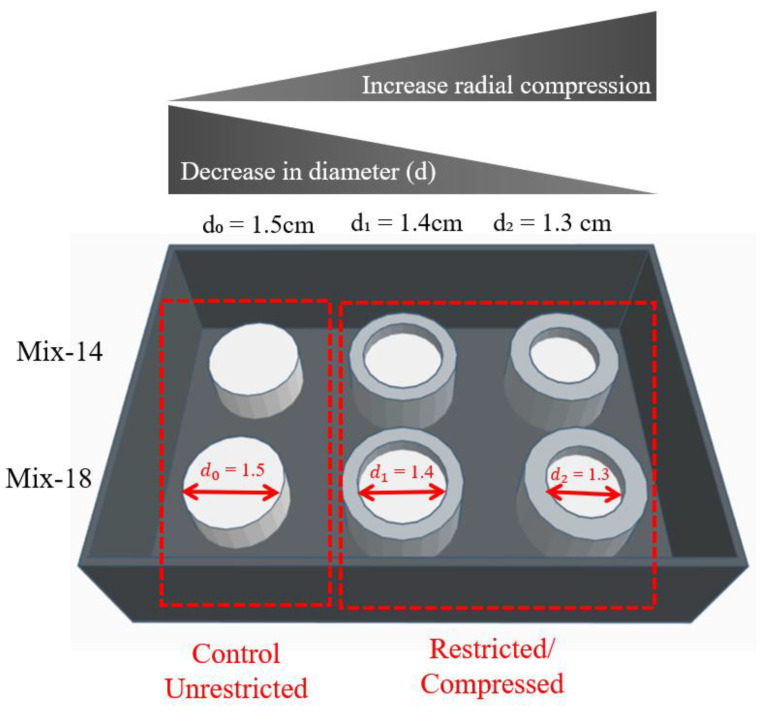
Diagram showing the setup used to build the custom designed radial compression apparatus. Left: unrestricted (control or d_0_ = 1.5 cm) shape memory polyurethane aerogels (SSMPA). Center and right: SSMPA with increased degrees of radial compression and final diameters of d_1_ = 1.4 cm and d_2_ = 1.3 cm, respectively. Top: SSMPA Mix-14. Bottom: SSMPA Mix-18.

The SSMPA used here were cylindrically shaped (d_0_ = 1.5 cm, in original form). The cylinders were sliced into thinner sections to create discs for PC12 cell plating. Controlled radial compression was achieved by placing the aerogels in a custom-designed, ring-shaped apparatus with an adjustable width, fabricated from Sylgard 184 Silicone Elastomer (Dow Corning, Midland, MI, USA). Custom-cut circular metal disks of the desired diameter (d_1_ = 1.4 cm and d_2_ = 1.3 cm) formed the base of the compression apparatus. Figure 1 shows a schematic diagram of the assembly used in this study to create the substrates needed.

### 2.3. Cell Culture

The PC12 neural cell line obtained from the American Type Culture Collection (ATCC, Manassas, VA, USA) was used in this investigation (see Figure S1) and has been the basis of several past publications by the authors [12,13,23,24]. Briefly, PC12 cells were primed in complete culture medium (RPMI 1640 medium supplemented with GlutaMax, HERPES buffer, 10% horse serum, 5% fetal bovine serum and penicillin/streptomycin) with nerve growth factor (NGF) for 8 days with the medium being replaced every 2–3 days. The cells were harvested by trypsin treatment and frozen in liquid nitrogen until the time of the experiment. After thawing, PC12 cells were plated on collagen I coated SSMPA Mix-14 and Mix-18. Prior to coating the aerogels with collagen, they were sterilized for 30 min with ultra-violet light (λ = 254 nm). After sterilization, the aerogel discs were coated with 4 µg of collagen per cm^2^, which translated into 0.05 mg/mL rat tail type I collagen (Invitrogen, Carlsbad, CA, USA) concentration. After 1 h of RT incubation, the excess solution was removed by rinsing the aerogels with serum free RMPI 1640 medium. The cells were seeded at a density of 1 × 10^4^ cells/cm^2^ and incubated in a tissue culture incubator (5% CO_2_ and 37 °C) for 24 h. The incubation period was previously optimized by the authors and reported elsewhere [12,13,23,24].

### 2.4. Scanning Electron Microscopy

SSMPA containing cells were prepared for Scanning Electron Microscopy (SEM) by fixation for 2 h in 2.5% glutaraldehyde (Tousimis, Rockville) in 0.1 M sodium phosphate buffer. After a series of washes in 0.1 M sodium phosphate buffer, the cells were further fixed for 30 min with an aqueous solution of 1% osmium tetroxide (OsO_4_) (Electron Microscopy Sciences, Hatfield, PA, USA). The cells were then dehydrated with a graded alcohol series (10%, 30%, 50%, 70%, 90% and 100% ethanol, 10 min for each wash). The samples were fully air dried and then sputter coated with a 10 nm film of gold-palladium using the Anatech Aummer 10.2 for 1–3 min at 3–5 mA (SemiStar Corp, Morgan Hill, CA, USA), as reported previously [12,13,23,24]. Phenom Pure Desktop SEM (PhenomWorld, Eindhoven, Netherlands) was used to image the samples using a backscatter electron detector (BSD), the high-resolution mode (10 kV) and a working distance of 5mm.

### 2.5. Cell Response Measurement

The SEM images were then analyzed with ImageJ, version 1.53d, an NIH (National Institutes of Health) open-source image analysis software (NIH, Bethesda, MD, USA). This software was used to quantify neurite length, the number of neurites per cell and cell area, as described in previous works [12,13,23,24]. Briefly, for each SEM image, a superimposed scale bar was used as the reference to calibrate the measurement tools in ImageJ. Neurite length was measured using the segmented line tool and using a series of segmented lines to follow the longest cytoplasmatic extension extending out of the cell body. The number of neurites per cell was calculated by measuring the number of cytoplasmatic extensions extending out of each cell body. Cell spreading was measured by using the free hand selection to outline each cell body. These three parameters were measured for each SSMPA (Mix-14 and Mix-18) in each radial compression state.

### 2.6. Pore Diameter Measurement

To measure the diameter of the superficially positioned pores at the surface of the aerogels, the aerogels were first sputter coated with a 10 nm film of gold-palladium and SEM imaging was performed. The NIH open source software ImageJ was used to measure the diameter of the pores at the surface of the aerogel. Each SEM image had an overlaid scale bar, which was used as reference to calibrate the measurement tools from ImageJ. The pore diameter was treated as the longest horizontal opening in each pore. The straight-line tool was used to measure the diameter of the pores. Three independent trials (N = 3) were performed for each SSMPA substrate and each final diameter. In each case, over 100 cells were investigated (*n* > 100).

### 2.7. Surface Roughness (Sa) Measurement

The surface roughness of all samples (control and increased degree of radial compression) was measured using a Profilm3D profilometer (Filmetrics Inc, San Diego, CA, USA) in an area of 200 µm × 200 µm. The arithmetic mean height indicated the surface roughness (Sa). Once again three independent measurements (N = 3) were performed for each SSMPA and each radial confinement condition.

### 2.8. Stiffness Measurement

The aerogel stiffness (Young’s modulus) was measured at RT by means of an ESM303 Series 5, digital Force Gauge (Mark-10 Corp, Copiague, NY, USA). The accessories used were the extension rod (G1024) and a compression plate (G1009) attached to the 25 N gauge. Experiments were performed at a compression rate of 0.5 mm/min. From the instrument, the Load (F in N) and travel (l in mm) was acquired and the Young’s modulus was calculated using the following formula as well as the sample’s thickness (l) and cross-sectional area (A):

$$\mathrm{Young}\prime \;\mathrm{smodulus}=\frac{\mathrm{stress}\;\left(\sigma \right)}{\mathrm{strain}\;\left(\varepsilon \right)}$$

where:

$$\mathrm{stress}\;\left(\sigma \right)=\frac{F\left(N\right)}{A\;\left(m^{2}\right)}$$

$$\mathrm{strain}\;\left(\varepsilon \right)=\frac{\Delta l\left(m\right)}{l\;\left(m\right)}$$

### 2.9. Statistical Analysis

Statistical analysis of the data was performed using a two-tailed Student’s *t*-test. Significance (*) is defined as *p* < 0.05, as in previous works [21,22]. For each parameter measured (neurite length, number of neurites per cell, cell area, pore diameter, Sa and Young’s modulus), the error bars represent the standard error of the mean. Each of the cell measurement results (neurite length, number of neurites per cell and cell area) were calculated using three independent trials (N = 3), with the measurements being carried out on at least 100 cells (*n* > 100).

To measure the pore diameter, three independent trials (N = 3) of three separate specimens were performed for each trial; more than 100 measurements (*n* > 100) were carried out. The average of each independent trial and the standard deviation were used for the results in Section 3.3. Sa averages were calculated based on three independent trials (N = 3) of three different specimens in each category. For each aerogel type, thirty different locations were sampled, and the results were averaged. The Young’s modulus was calculated for each type of aerogel, and, once again, three independent measurements were performed in each case (N = 3).

## 3. Results

### 3.1. Effect of Radial Compression on the SSMPA Properties

SSMPA properties, including pore diameter, Sa and Young’s modulus, were measured to determine correlations with the response of neural cells plated on these SSMPA. These properties were measured for each SSMPA (Mix-14 and Mix-18), which were subjected to different degrees of radial compressions (unrestricted or control, d_0_ = 1.5 cm, and increasing degrees of radial compression, d_1_ = 1.4 cm and d_2_ = 1.3 cm,) and the details have been reported below.

#### 3.1.1. Pore Diameter

**Figure 2:**
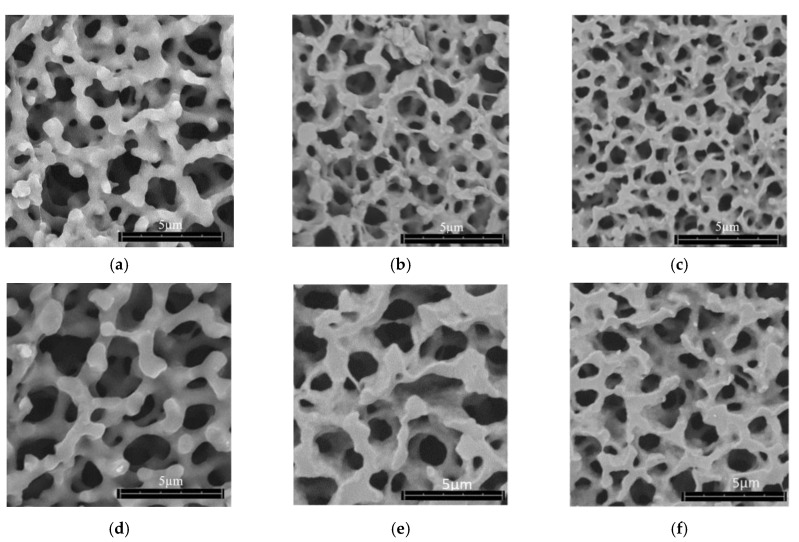
SEM images of SSMPA unrestricted (control or d_0_ = 1.5 cm) and different degrees of radial compression (d_1_ = 1.4 cm and d_2_ = 1.3 cm). SEM images of bare aerogels (i.e., not coated with collagen nor plated with cells). (**a**–**c**) SSMPA Mix-14 and (**d**–**f**) SSMPA Mix-18; (**a**,**d**) unrestricted (control or d_0_ = 1.5 cm); (**b**,**e**) lesser degree of radial compression (d_1_ = 1.4 cm); (**c**,**f**) higher degree of radial compression (d_2_ = 1.3 cm).

Figure 2 shows representative SEM images of each aerogel type with increasing degrees of radial compression (d_0_ = 1.5 cm, d_1_ = 1.4 cm, d_2_ = 1.3 cm). The average pore size of unrestricted (control, d_0_ = 1.5 cm) Mix-14 aerogel was 0.77-fold smaller than that of Mix-18 unrestricted (control or d_0_ = 1.5 cm) (Figure 2a,d). For both aerogels, controlled radial compression led to a decrease in pore size by ~0.9-fold between unrestricted (control, d_0_ = 1.5 cm) and lesser degree of radial compression (d_1_ = 1.4 cm). A decrease of ~0.76-fold was observed between the lesser and higher degree of radial compression (d_1_ = 1.4 cm and d_2_ = 1.3 cm). The measured pore diameters have been summarized in Table 1, showing a reduction in pore size as the degree of radial compression is increased.

#### 3.1.2. Surface Roughness (Sa)

**Figure 3:**
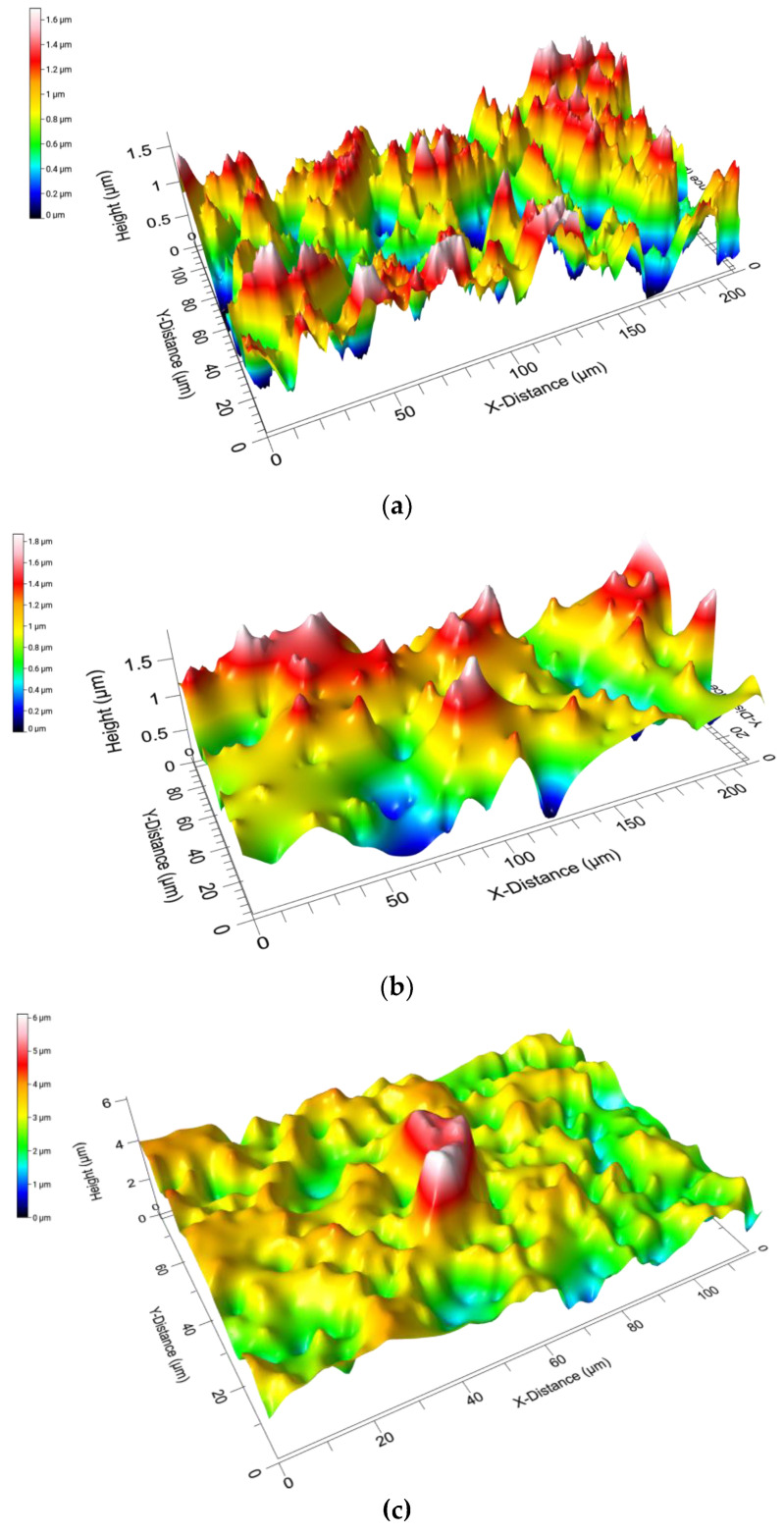
3-D images of the surface roughness of unrestricted (control or d_0_ = 1.5 cm) aerogels. (**a**) 3-D image of the SSMPA Mix-14 and (**b**) 3-D image of the SSMPA Mix-18. (**c**) 3-D image of SSMPA and PC12 cell growing on the aerogel. The color scale bar represents the *Z* axis throughout the surface of the aerogels.

The effect of controlled radial compression on the Sa of Mix-14 and Mix-18 aerogels was evaluated. Table 2 shows the Sa values for Mix-14 and Mix-18 with increasing radial compression (d_0_ = 1.5 cm, d_1_ = 1.4 cm, d_2_ = 1.3 cm). Figure 3 shows 3-D images of the Sa of unrestricted (control or d_0_ = 1.5 cm) Mix-18 and Mix-14, with a color scale bar demonstrating the different height (*z* axis) throughout the surface. Mix-18 inherently has a higher Sa value compared to Mix-14 as evidenced by the data shown in Table 2 and Figure 3. SSMPA Mix-14 had a ~0.87-fold lower Sa compared to Mix-18 in the unrestricted (control or d_0_ = 1.5 cm) state. As the degree of radial compression was increased, the Sa for both aerogel types decreased. SSMPA Mix-14 showed a decrease of ~0.86-fold between unrestricted (control-d_0_ = 1.5 cm) and the lesser degree of radial compression (d_1_ = 1.4 cm), as well as the lesser and higher degree of radial compression (d_1_ = 1.4 cm and d_2_ = 1.3 cm). SSMPA Mix-18 showed a decrease of ~0.92-fold in Sa between unrestricted (control-d_0_ = 1.5 cm) and lesser degree of radial compression and a decrease of ~0.79-fold between lesser and higher degree of radial compression (d_1_ = 1.4 cm and d_2_ = 1.3 cm).

#### 3.1.3. Young’s Modulus

The last aerogel property measured was the stiffness of the substrate. Table 3 shows the change in stiffness of each aerogel type with increasing radial compression. In their relaxed unrestricted forms, Mix-14 and Mix-18 had distinctly different stiffness values (255 vs. 182 kPa), with Mix-18 being less stiff. However, the controlled radial compression did not affect the aerogel stiffness substantially, though small changes in Young’s modulus (increase) were observed for both aerogel types.

#### 3.1.4. Comparison of Pore Diameter and Surface Roughness

**Figure 4:**
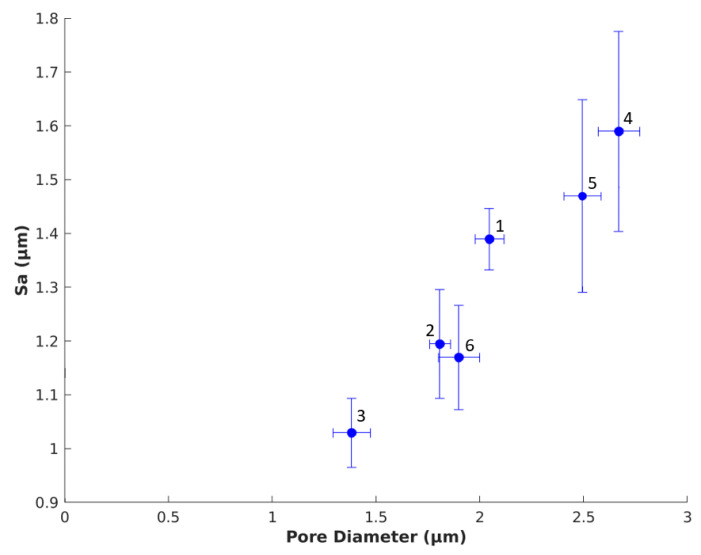
Relationship between pore diameter and Sa. Graph demonstrating the effect of change in pore diameter on surface roughness (Sa). As the pore diameter decreases (due to increased radial compression), the surface roughness also decreases. Labels 1–6 correspond to those identified in Table 1 and Table 2.

Figure 4 shows the relationship between the pore diameter and the Sa for SSMPA Mix-14 and Mix-18 subjected to different degrees of radial compression (d_0_ = 1.5 cm, d_1_ = 1.4 cm, d_2_ = 1.3 cm). As indicated earlier, for both SSMPA Mix-14 and Mix-18, pore diameter and Sa decreased with increased radial compression. There is a decrease in pore diameter between 0.7-fold and 0.96-fold, showing that increased radial compression significantly reduces the SSMPA pore diameter. Moreover, with increased radial compression, the Sa also decreased by 0.86–0.97-fold.

### 3.2. Impact of SSMPA Properties on Cell Shape and Neurite Extension

The impact of SSMPA substrate properties on PC12 neuronal cell response was evaluated and is discussed below. The cell properties that were investigated and that have been fully characterized included neurite length, the number of neurites per cell and cell body area. SSMPA Mix-14 and Mix-18 with increasing amounts of radial compression (d_0_ = 1.5 cm, d_1_ = 1.4 cm, d_2_ = 1.3 cm) altered the behavior of the cells, and the results are presented below.

#### 3.2.1. Neurite Length

**Figure 5:**
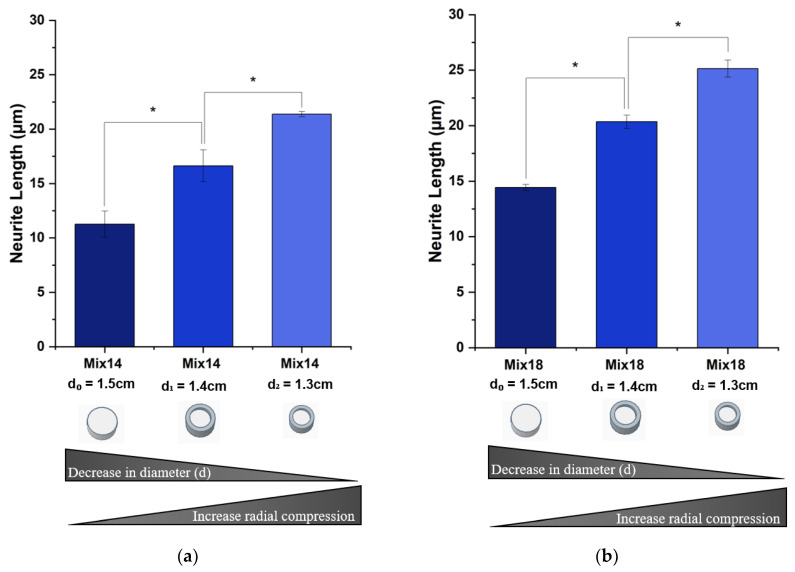
Neurite length averages of PC12 cells plated on SSMPA. (**a**) Mix-14; (**b**) Mix-18 with a different extent of radial compression (the highest radial compression is achieved for a smaller diameter aerogel). For each condition, averages were obtained from the results of three independent trials (N = 3) in which over 100 measurements (*n* > 100) were performed. Error bars correspond to the standard error of the mean. Asterisks (*) denote values that are statistically significant (*p* < 0.05). The results indicate that, for both Mix-14 and Mix-18, radial compression significantly increased neurite length by 1.8-fold and 2-fold, respectively.

Figure 5 shows the neurite length in SSMPA Mix-14 (Figure 5a) and Mix-18 (Figure 5b) with increasing degrees of radial compression (d_0_ = 1.5 cm, d_1_ = 1.4 cm, d_2_ = 1.3 cm). Increasing the degree of radial compression for both aerogel types resulted in an increase in the average neurite length after a 24 h period of incubation. In the case of Mix-18 (Figure 5b), the average neurite length was greater than what was measured on Mix-14, almost double the length (Figure 5a). When compared to unrestricted (control d_0_ = 1.5 cm) and the higher degree of radial compression (d_2_ = 1.3 cm), the maximum neurite length increase was ~1.8-fold for cells plated on SSMPA Mix-18, while for Mix-14 the increase was ~2-fold.

#### 3.2.2. Number of Neurites per Cell Body

**Figure 6:**
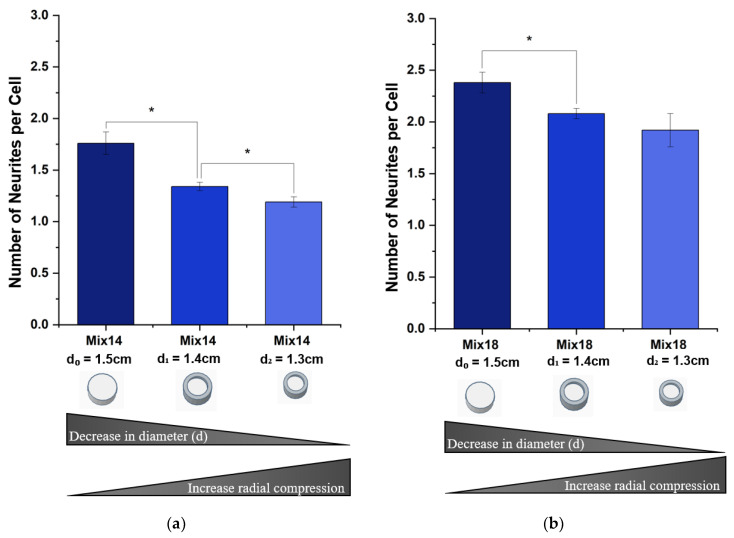
Average number of neurites per cell. For both SSMPA (**a**) Mix-14 and (**b**) Mix-18, compression decreased the number of processes extended by the cells. However, this decrease was more pronounced for Mix-14 with a decrease of ~1.4-fold, while Mix-18 shows a decrease of ~1.2-fold. Averages were measured with three independent (N = 3) trials with over 100 measurements (n > 100). Error bars represent the standard error of the mean within these different trials. Asterisks (*) denote values that are statistically significant (*p* < 0.05).

Figure 6 shows the number of neurites extended per neuronal cell with increasing degrees of radial compression (d_0_ = 1.5 cm, d_1_ = 1.4 cm, d_2_ = 1.3 cm) for both types of aerogels Mix-14 (Figure 6a) and Mix-18 (Figure 6b). The number of neurites extended per neuronal cell decreased with increasing degrees of radial compression for both types of aerogels. The degree of decrease is more pronounced for Mix-14 (Figure 3a) than for Mix-18 (Figure 3b). The maximum decrease in neurites per neuronal cell (d_2_ = 1.3 cm), when compared to the unrestricted (control or d_0_ = 1.5 cm), is a ~1.4-fold decrease in Mix-14, while the decrease was ~1.2-fold in Mix-18.

#### 3.2.3. Cell Body Area

**Figure 7:**
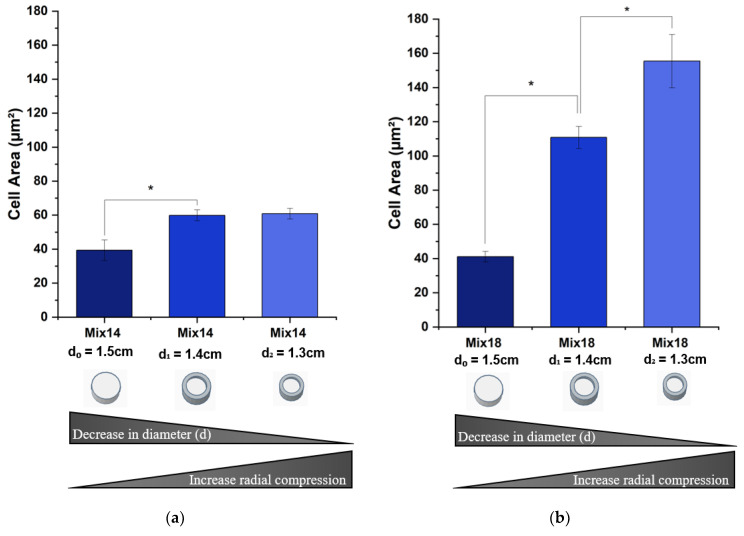
Comparison of cell body area on Mix-14 and Mix-18 aerogels. In the relaxed form, the PC12 cells plated on (**a**) Mix-14 had similar areas to those measured on (**b**) Mix-18. As the substrate received an increasing degree of radial compression, the cell area was affected more significantly for cells plated on Mix-18. Average was calculated from three independent trials (N = 3) with over 100 measurements (n > 100) each. Error bars represent the standard error of the mean. Asterisks (*) denote values that are statistically significant (*p* < 0.05).

Figure 7 shows the cell body area measurements under different degrees of radial compression collected from cells cultured on SSMPA Mix-14 and Mix-18. The results indicate that, for both substrate types, the cell body area increased with an increased degree of radial compression. However, this increase was much more pronounced on Mix-18. A ~4-fold increase between unrestricted (control-d_0_ = 1.5 cm) and the higher degree of radial compression (d_1_ = 1.4 cm) was observed for Mix-18 (Figure 7b). To the contrary, Mix-14 showed a ~1.5-fold increase between unrestricted (control-d_0_ = 1.5 cm) and the higher degree of radial compression (d_2_ = 1.3 cm) (Figure 7a).

### 3.3. Nerve Response to Sa

**Figure 8:**
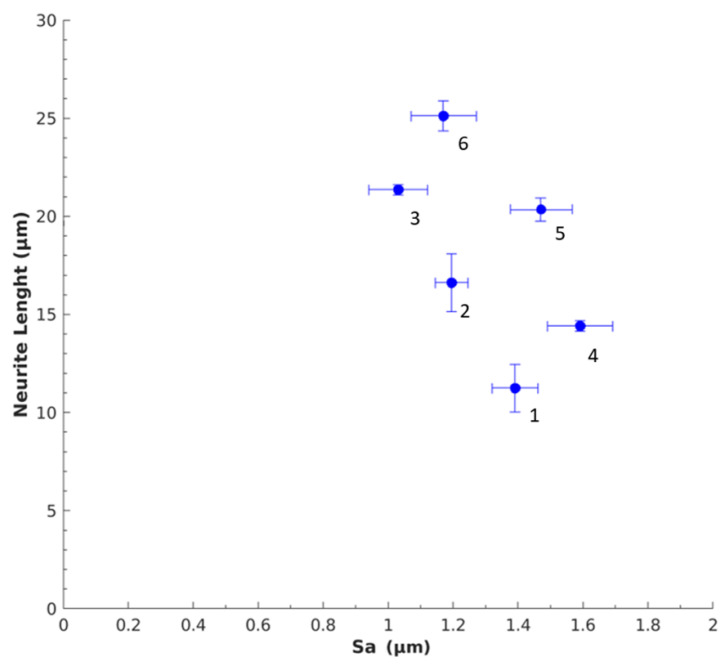
Relationship between Sa and neurite length. As the degree of surface roughness increased, the average neurite length decreased. Labels correspond to those in Table 1 and Table 2.

**Figure 9:**
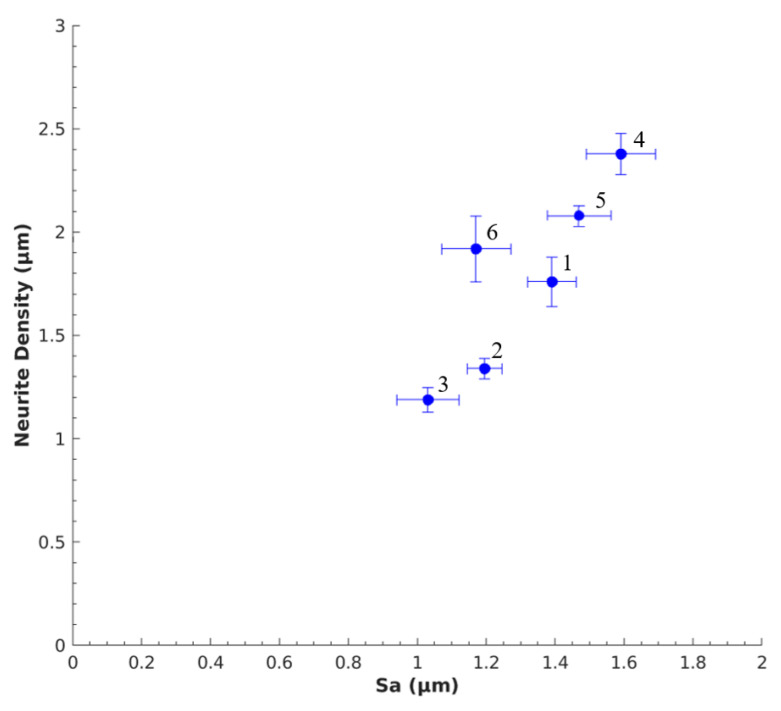
Relationship between Sa and number of neurites per cell body. As the degree of surface roughness increased, the number of neurites per cell body also increased. Labels correspond to those indicated in Table 1 and Table 2.

The neuronal response to Sa was compared to get an overall understanding of the impact of Sa on PC12 cells. Figure 8 shows the relationship between neurite length and Sa for SSMPA Mix-14 (1,2,3) and Mix-18 (4,5,6), as labeled in Table 2. In Figure 9, the correlation between the number of neurites per cell body and Sa for both types of aerogels is provided. Greater surface roughness (Sa) led to shorter neurite lengths for both Mix-14 and Mix-18 (Figure 8), while it led to an increase in neurite density (Figure 9). An overall increase in neurite length of 1.05–1.28-fold was observed for each aerogel type (Figure 8). On the other hand, an overall decrease in neurite density of 0.76–0.92-fold was observed for each aerogel type with decreasing S_a_ (Figure 9).

## 4. Discussion

PC12 neuronal cells are a model of peripheral neurons because they are derived from a tumor of the peripheral nervous system and because, under in vitro conditions, they extend long cellular processes. As such, PC-12 cells are accepted as a good model for nerve regeneration in studies investigating the effect of different in vitro conditions on the length of neurites. In previous studies, we have shown that aerogel substrates enhance neurite outgrowth [12,13,23,24]. The aerogels investigated in our earlier studies include polymer-crosslinked silica aerogel (PCSA) [11,12,14,19,20] and carbon aerogel (CA) [13], both of which are significantly stiffer than the aerogels investigated in this study [12,13,23]. Our earlier studies showed that, compared to PC12 plated on glass or plastic substrates, PC12 cells plated on aerogels extended longer neurite lengths. This result served as a strong motivator to explore the response of PC12 cells plated on other aerogel types exhibiting various properties. The SSMP aerogels used here offered bulk and surface properties distinctly different from those studied previously [12,13,23,24] and hence were chosen for this investigation. The use of SSMP aerogels as a neuronal scaffold has not been investigated previously. The results of the biomedically relevant investigation reported here show that SSMP aerogels have biomedical promise as materials for nerve regeneration because the length of the neurites extended by PC12 cells grown on SSMP aerogels is greater than that of PC12 cells grown on polymer-crosslinked silica aerogels and carbon aerogels, which were used in our previous studies [12,13,23].

The pore size, and hence the surface roughness (Sa), of SSMP aerogels could be incrementally modified by varying the conditions used to prepare the aerogels, and these two parameters could be further modified by applying controlled amounts of radial pressure. Interestingly, our Young’s modulus measurements show that these changes in pore size and Sa were accomplished without significantly affecting the stiffness of the aerogel. This enabled us to evaluate PC12 neurite extension as a function of two key parameters of SSMP aerogels, pore diameter and Sa, while keeping the surface stiffness constant. The results show that SSMPA Mix-14 and Mix-18 not only supported PC12 neuronal cell attachment and neurite outgrowth but also affected cell body area, neurite length and neurite density. Using SEM imaging and profilometry, we demonstrated that radially compressing SSMP aerogels in our custom-built apparatus uniformly reduced pore diameters. Cells in general, and particularly neurons such as PC12 cells, are very sensitive to surface roughness (Sa) [32,33].

First, we compared the behavior of PC12 cells on SSMPA Mix-14 and Mix-18 in their relaxed, unrestricted (control) state. Mix-14 and Mix-18 differ in Sa (1.39 vs. 1.59 µm), Young’s modulus (255 vs. 182 KPa) and pore size (3.05 vs. 2.64 µm) in an unrestricted state (control-d_0_ = 1.5 cm). The cell body area was not affected by the inherent differences between Mix-14 and Mix-18 (Figure 7). However, PC12 cells extended longer neurites (Figure 5) and had a greater neurite density on Mix-18 when compared to Mix-14 (Figure 6).

When Mix-14 and Mix-18 were compressed to a final diameter of d_1_ = 1.4 cm, the cell body area, neurite length and neurite density of PC12 cells were all affected to various degrees. Compared to the uncompressed aerogels, this compression increased significantly the cell area on Mix-18 (2.7-fold) and also on Mix-14 (1.5-fold), albeit to a lesser degree. The compression also increased neurite length on both Mix-14 and Mix-18 to a similar degree (1.47-fold and 1.41-fold, respectively). Neurite density, however, decreased nearly equally on Mix-14 and Mix-18 (0.76-fold and 0.87-fold, respectively) compared to the uncompressed aerogels.

When Mix-14 and Mix-18 were compressed to a final diameter of d_2_ = 1.3 cm, the changes observed at d_1_= 1.4 cm compared to uncompressed were more pronounced. The cell area increased 1.02-fold and 1.40-fold on Mix-14 and Mix-18, respectively. Neurite length also further increased 1.89-fold and 1.74-fold on Mix-14 and Mix-18, respectively, while neurite density continued to decrease steadily 0.68-fold and 0.81-fold on Mix-14 and Mix-18, respectively.

The conditions tested in this investigation demonstrate that increased surface roughness and pore size induce PC12 cells to assume morphological features characteristic of differentiated neurons; that is, an extended cell body from which originate fewer but longer neurites. This finding supports the notion that cells are very sensitive to surface roughness [32,34]. However, finding difference with the studies of Brunetti et al. (2010) and Pennisi et al. (2011), which found that increased surface roughness had a somewhat deleterious effect on cells, our results indicate a positive effect represented by increased differentiation. This could be due to the fact that the surfaces used in the studies of Brunetti et al. (2010) and Pennisi et al. (2011) were metallic (gold or platinum) and non-porous, which differs from the porous, organic aerogels used here.

Therefore, our results demonstrate that SSMPA aerogels have a great potential for biomedical applications such as nerve regeneration. This potential is due to the facts that SSMPA aerogels are materials with a surface topography that includes pores in addition to raised features (mounds) and that their chemistry, unlike that of metallic surfaces, appears to be well tolerated by cells. In addition, the Sa and pore size of SSMPA aerogels can be tuned after their synthesis by subjecting the aerogels to radial compression. Future studies will thoroughly investigate the relationship between SSMPA aerogel chemistry, Sa and pore size on the one hand and neural differentiation on the other hand to identify the optimal parameter for neural differentiation.

In conclusion, our study shows that the surface roughness and pore size of SSMPA aerogels can be tuned after their synthesis by radial compression to values supporting neuronal differentiation. These characteristics make SSMPA aerogels a potential material for the repair of nerve injuries.

## Figures and Tables

**Table 1:** Effect of radial compression on SSMPA pore size.

Aerogel Type	Degree of Radial Compression	Pore Diameter (µm)	Label
Mix-14	d_0_ = 1.5	2.05 ± 0.16	1
	d_1_ = 1.4	1.81 ± 0.10	2
	d_2_ = 1.3	1.38 ± 0.06	3
Mix-18	d_0_ = 1.5	2.67 ± 0.19	4
	d_1_ = 1.4	2.50 ± 0.18	5
	d_2_ = 1.3	1.90 ± 0.10	6

**Table 2:** Surface roughness (Sa) of each SSMPA used in this study.

Aerogel Type	Degree of Radial Compression	Sa (µm)	Label
Mix-14	d_0_ = 1.5	1.39 ± 0.17	1
	d_1_ = 1.4	1.20 ± 0.25	2
	d_2_ = 1.3	1.03 ± 0.12	3
Mix-18	d_0_ = 1.5	1.59 ± 0.17	4
	d_1_ = 1.4	1.47 ± 0.23	5
	d_2_ = 1.3	1.17 ± 0.10	6

**Table 3:** Young’s modulus (kPa) for each SSMPA type and condition.

Aerogel Type	Degree of Radial Compression	Young’s Modulus (kPa)	Label
Mix-14	d_0_ = 1.5	255 ± 33	1
	d_1_ = 1.4	269 ± 65	2
	d_2_ = 1.3	298 ± 19	3
Mix-18	d_0_ = 1.5	182 ± 18	4
	d_1_ = 1.4	199 ± 78	5
	d_2_ = 1.3	203 ± 83	6

## Supplementary Materials

The following are available online at https://www.mdpi.com/2073-4360/12/12/2995/s1, Figure S1: SEM image of PC12 cell with lines representing the neurite length and orientation measured using ImageJ. The yellow line represents the measurement of neurite length. The blue line shows the cell body area measurement.
