# Supplementary material for: Nerve Response to Superelastic Shape Memory Polyurethane Aerogels
Source: Polymers (Basel). 2020 Dec 15;12(12):2995. doi: 10.3390/polym12122995 (PMC7765513; doi:10.3390/polym12122995)
Supplement: Supplementary file 1 [file polymers-12-02995-s001.pdf]

**Supplementary Material:**

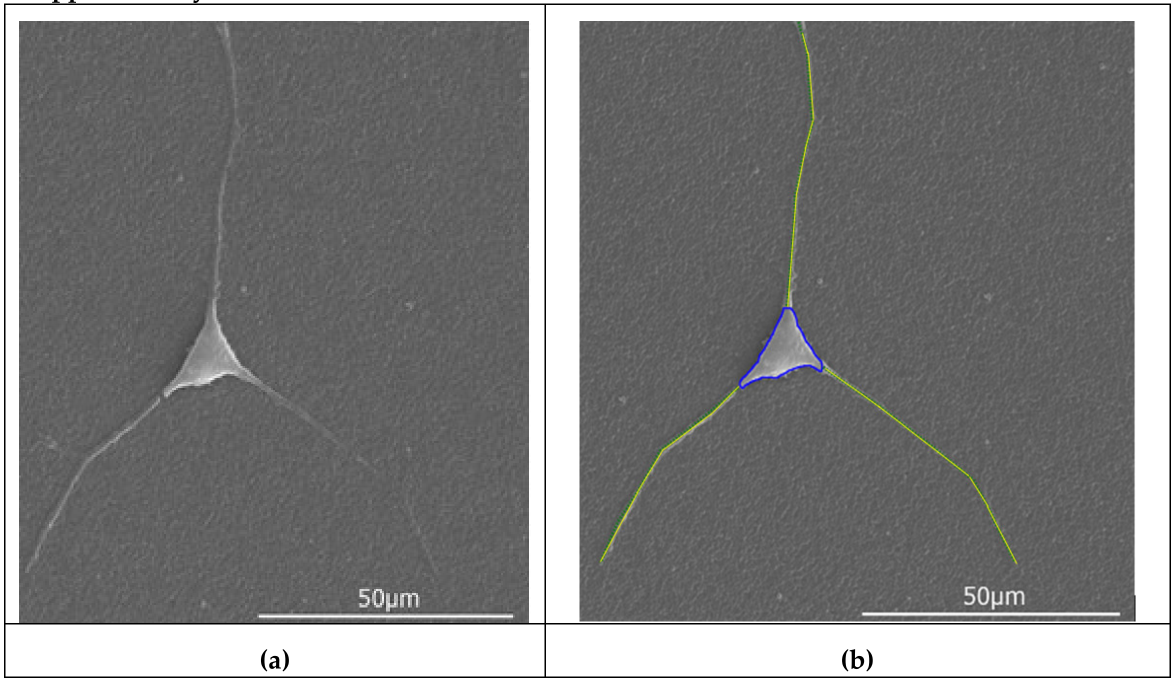


| | |
|---|---|
| **(a)** | **(b)** |

**Figure S1: SEM image of PC12 cell with lines representing the neurite length and orientation measured using ImageJ. The yellow line represents the measurement of neurite length. The blue line shows the cell body area measurement.**
